# Use of Mobile Phone App Interventions to Promote Weight Loss: Meta-Analysis

**DOI:** 10.2196/17039

**Published:** 2020-07-22

**Authors:** Md Mohaimenul Islam, Tahmina Nasrin Poly, Bruno Andres Walther, Yu-Chuan (Jack) Li

**Affiliations:** 1 Graduate Institute of Biomedical Informatics College of Medical Science and Technology Taipei Medical University Taipei Taiwan; 2 International Center for Health Information Technology (ICHIT) Taipei Medical University Taipei Taiwan; 3 Research Center of Big Data and Meta-Analysis Wan Fang Hospital Taipei Medical University Taipei Taiwan; 4 Department of Biological Sciences National Sun Yat-Sen University Kaohsiung Taiwan; 5 Department of Dermatology Wan Fang Hospital Taipei Medical University Taipei Taiwan

**Keywords:** mobile app, mHealth, obesity, physical activity, weight gain prevention

## Abstract

**Background:**

Obesity and lack of physical activity are major health risk factors for many life-threatening diseases, such as cardiovascular diseases, type 2 diabetes, and cancer. The use of mobile app interventions to promote weight loss and boost physical activity among children and adults is fascinating owing to the demand for cutting-edge and more efficient interventions. Previously published studies have examined different types of technology-based interventions and their impact on weight loss and increase in physical activity, but evidence regarding the impact of only a mobile phone app on weight loss and increase in physical activity is still lacking.

**Objective:**

The main objective of this study was to assess the efficacy of a mobile phone app intervention for reducing body weight and increasing physical activity among children and adults.

**Methods:**

PubMed, Google Scholar, Scopus, EMBASE, and the Web of Science electronic databases were searched for studies published between January 1, 2000, and April 30, 2019, without language restrictions. Two experts independently screened all the titles and abstracts to find the most appropriate studies. To be included, studies had to be either a randomized controlled trial or a case-control study that assessed a mobile phone app intervention with body weight loss and physical activity outcomes. The Cochrane Collaboration Risk of Bias tool was used to examine the risk of publication bias.

**Results:**

A total of 12 studies involving a mobile phone app intervention were included in this meta-analysis. Compared with the control group, the use of a mobile phone app was associated with significant changes in body weight (−1.07 kg, 95% CI −1.92 to −0.21, *P*=.01) and body mass index (−0.45 kg/m2, 95% CI −0.78 to −0.12, *P*=.008). Moreover, a nonsignificant increase in physical activity was observed (0.17, 95% CI −2.21 to 2.55, *P*=.88).

**Conclusions:**

The findings of this study demonstrate the promising and emerging efficacy of using mobile phone app interventions for weight loss. Future studies are needed to explore the long-term efficacy of mobile app interventions in larger samples.

## Introduction

Overweight (BMI ≥25 kg/m^2^), obesity (BMI ≥30 kg/m^2^), and physical inactivity are major preventable public health problems that are linked to increased risks of chronic diseases such as diabetes, high blood pressure, heart disease, and cancer [1]. In 2016, over 1.9 billion (39%) and over 650 million (13%) adults older than 18 years were overweight and obese, respectively, and the prevalence of overweight and obesity has nearly tripled since 1980 [2]. Reducing weight and improving physical activity are thus important priorities to minimize the burden associated with overweight and obesity-related comorbidities. A high number of studies have already demonstrated that a change in lifestyle can help reduce and maintain weight [3,4]. However, for many people, it is very difficult to change their lifestyle and maintain weight loss [5]. Therefore, an intervention that helps to motivate people to undertake a change in lifestyle, offers pragmatic goal settings, and offers feedback on activity rates can help maintain weight loss and greatly increase physical activity [6,7].

The mobile phone has become a very important medium of communication throughout the world [8], and approximately 75% of adults have used different kinds of mobile interventions [9,10]. Therefore, the use of a mobile phone intervention could be a promising approach for disease management and prevention that has a huge potential to reach out to the vast majority of the population [11]. Researchers have been using mobile phone interventions to support behavioral change by providing more interactive and timely access to relevant information and delivering context-specific prompt assistance [12]. Recently, the use of mobile apps has led to notable success in obesity control, weight reduction, physical activity increase, and quality of life improvement [13]. App-based interventions have already been shown to be cost-effective and to reduce the immense barriers associated with more traditional approaches. However, the effectiveness and success of a mobile app intervention also relies on how the intervention has been developed. A mobile app intervention delivery system has the capacity to reach each participant effectively and efficiently, and it is thus an innovative way to manage weight and increase physical activity.

The use of a mobile app is a rapidly expanding area of research for disease management and behavioral change. Therefore, the purpose of this systematic review and meta-analysis was to evaluate the current evidence for the feasibility of a mobile phone intervention. Our study updates and extends the scope of a prior systematic review and meta-analysis in three ways [14]. First, we included two more randomized controlled trials than the previous review. Second, we provided more subgroup analyses than the previous review. Third, we extended the sample size and characteristics, such as age, gender, geographic region, and features of the intervention, to evaluate mobile app efficacy for weight loss and increased physical activity.

## Methods

### Guidelines

This systematic review was conducted in accordance with the Meta-Analysis of Observational Studies in Epidemiology guidelines [15] and the Preferred Reporting Items for Systematic Reviews and Meta-Analyses standard (Multimedia Appendix 1) [16].

### Literature Search

PubMed, Google Scholar, Scopus, EMBASE, and the Web of Science electronic databases were searched for studies published between January 1, 2000, and April 30, 2019, without language restrictions. The search was conducted by two experts (MMI and TNP) using combinations of relevant search terms and Boolean operators as follows: “mobile apps” AND “weight loss” OR “weight control” OR “obesity” OR “BMI” OR “body mass index” (Multimedia Appendix 2). There was no language or data restriction for the initial search. However, we did not consider any gray literature for unpublished studies (abstracts and conference proceedings) in the initial search. Such unpublished studies were not considered because they did not pass a proper peer-review process. We then used EndNote X7 (Thomson Reuters) to remove any duplicate publication.

### Other Resources Search

We carefully checked all the retrieved systematic reviews and meta-analyses in order to find further relevant studies.

### Eligibility Criteria

Two experts (MMI and TNP) independently screened all the titles and abstracts to find the most appropriate full-text studies for inclusion. They then further screened all full-text studies for quantitative synthesis of evidence and recorded the inclusion and exclusion criteria. Any disagreements over the inclusion and exclusion criteria that arose during this stage were subsequently resolved by the main investigator (YCL) of this study. We considered all studies if they met the following criteria: (1) published in English; (2) reported a mobile app intervention for change in body weight, BMI, or waist circumference; and (3) reported a mobile app for weight loss among children and adults compared with a control group.

Studies were excluded if they met the following criteria: (1) review or methodology study, short communication, or letter to the editor; (2) case report or case series; (3) no control group; (4) outcomes of interest were other diseases except for a diagnosis of obesity; and (5) other types of mobile phone interventions like text messaging.

### Data Extraction

A predefined standard procedure was used to retrieve all the information from the included studies by the same two authors (MMI and TNP). They also used the Review Manager software (RevMan-5) to check the accuracy of the included studies. They collected the following information from all the included studies: (1) *methods*: number of studies, number of patients, age of participants, gender of participants or patients, study period, inclusion and exclusion criteria, and intervention and follow-up duration; (2) *results*: study characteristics, target group outcome, intervention characteristics, type of intervention, change of participant’s behavior, mean changes from baseline, variation that was reported as SD or 95% CI, and bias assessment; and (3) *discussion*: main findings, suggestions, intended recommendations, and limitations.

### Study Quality Assessment

The methodological quality of the included observational design studies was evaluated in accordance with the Cochrane Handbook for Systematic Reviews of Intervention [17]. This guideline is used to evaluate how well-randomized controlled trials were conducted to avoid bias based on a total of seven criteria. The following elements are reported in these guidelines: random sequence generation, allocation concealment, blinding of participants and personnel, blinding of outcome assessment, incomplete outcome data, selective reporting, and other bias. Therefore, the total score was used to assess the quality of the included studies as low, uncertain, or high risk.

### Statistical Analysis

The meta-analysis was performed to evaluate mobile app interventions for weight loss. A random-effect model was used to obtain an overall effect size. We calculated the standardized mean difference between the experimental group (mobile app) and control group using the mean, standard deviation, and total number of individuals. Moreover, an effect size and standard error for outcome (weight loss, BMI, and physical activity) were also calculated. We calculated the effect size with a 95% CI, and statistical significance was considered at a *P* value <.05. The chi-square (Q) statistic and I^2^ were also calculated to determine the sources of heterogeneity between the studies. If the *P* value of the chi-square test was >.05, the findings of the study were considered to be due to chance. Homogeneity between the studies was determined if the *P* value was >.05. The I^2^ value quantitatively determines heterogeneity. The heterogeneity between studies was categorized as very low, low, medium, and high if the I^2^ values were 0%-25%, 25%-50%, 50%-75%, and more than 75%, respectively [18]. A random-effect model was utilized because it is the appropriate model to calculate the effect size if the included studies are heterogeneous, and for homogenous studies, fixed-effect models are appropriate [19]. In addition, a funnel plot was generated to assess publication bias, and it was evaluated by the Egger method. All analyses were conducted using Comprehensive Meta-Analysis software (CMA) Version 2.

## Results

### Study Selection

**Figure 1:**
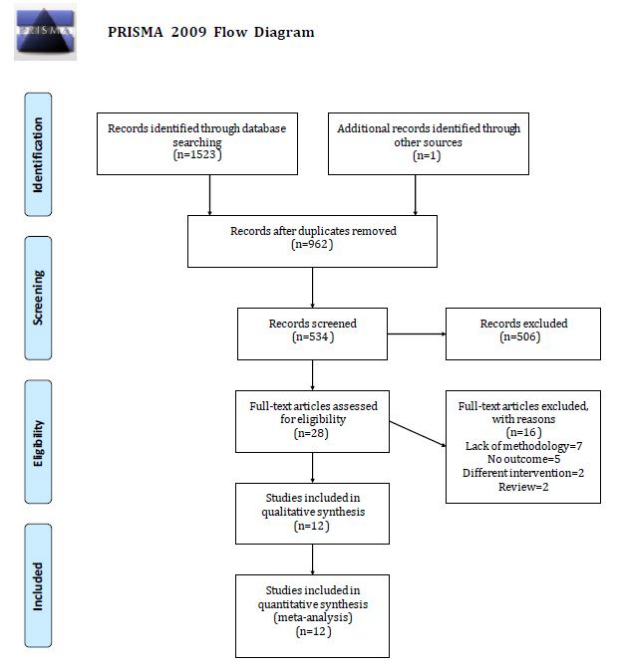
Flow chart of the study search and selection.

The literature search of the electronic databases yielded 1523 studies. After our review of the titles and abstracts, a total of 1495 studies were excluded either because of duplication or because of the lack of adherence to our topic. Consequently, only 28 studies underwent full-text review. We also checked their reference lists for further relevant studies and retrieved one additional study. However, 16 more studies were then excluded because they did not meet the inclusion criteria mentioned previously. Finally, a total of 12 studies were included in our meta-analysis [13,20-30]. The flow chart of our systematic literature search is presented in Figure 1.

### Study Characteristics

The studies included in our meta-analysis were 11 randomized controlled trials and 1 case-control study (Table 1). The publication year ranged from 2010 to 2019. The studies included child and adult participants, with a total of 792 experimental participants and 799 controls. Five studies were published in Australia, four in North America, two in Europe, and one in Asia. A total of 1714 participants were included, and the sample size ranged from 35 to 361. Most of the participants were female. The proportion of female participants ranged from 85% to 100%, and the mean age of the participants ranged from 12.7 to 44.9 years. The follow-up period of the included studies ranged from 6 weeks to 9 months. Moreover, age, gender, marital status, and education level were used as baseline variables, and mobile apps, email, etc were considered as study interventions (Table 2).

**Table 1:** Characteristics of the studies included in the meta-analysis.

First author (year)	Country	Study design	Study sample	Male, %	Age (years), mean	Study duration	Inclusion criteria	Exclusion criteria	Outcomes
Patel (2019)	USA	RCT^a^	100	16	42.7	3 months	Age 21-65 years with BMI 25-45 kg/m^2^, and willingness to reduce weight through dietary change. Availability of an iPhone or Android smartphone and personal email address, and ability to read and write in English.	Enrollment in other weight loss programs, use of MyFitnessPal to track diet in the past 6 months, loss of ≥10 lb, or use of weight loss medication in the past 6 months. Moreover, pregnancy and disorders, such as cancer, eating disorders, uncontrolled hypertension, diabetes mellitus, cardiovascular events, and congestive heart failure.	Weight and BMI
Farinelli (2016)	Australia	RCT	258	40.7	28.1	9 months	Age 18-35 years, BMI 25.0-31.9 kg/m^2^ or 23.0-24.9 kg/m^2^ with reported weight gain greater than 2 kg over the previous 12 months. Fruit intake of less than two servings daily, vegetable intake of less than five servings daily, and SSB^b^ intake of at least 1 L weekly. Energy-dense meals prepared away from home more than once per week. Owning a mobile phone capable of receiving text messages and having access to the internet at least once a week.	Pregnancy or plan for pregnancy within the next 9 months, enrollment in another mobile app–based weight loss program, weight reduction more than 10 kg voluntarily in the past 3 months, taking medications that cause more than 2 kg of weight gain, medical conditions that preclude following dietary or physical recommendations, history of disorders like eating disorders, and inability to read or write in English.	Weight, BMI, and PA^c^
Partridge (2015)	Australia	RCT	250	38	27.2	9 months	BMI 25.0-31.9 kg/m^2^ or 23.0-24.9 kg/m^2^ with reported weight gain greater than 2 kg over the previous 12 months, fruit intake of less than two servings daily, vegetable intake of less than five servings daily, SSB intake of at least 1 L weekly, energy-dense meals prepared away from home more than once per week, etc.	Pregnancy or plan for pregnancy within the study period, enrollment in other mobile app–based weight loss programs, reduction in weight greater than 10 kg in the past 3 months, use of medications that help to gain weight greater than 2 kg, other medical conditions that preclude following dietary or physical activity recommendations, and inability to speak English.	Weight, BMI, MPA^d^, and VPA^e^
Laing (2014)	USA	RCT	212	27	43.3	6 months	Age ≥18 years, BMI ≥25 kg/m^2^, and smartphone ownership.	Current, planned, or previous pregnancy within the last 6 months, hemodialysis, life expectancy less than 6 months, lack of interest in weight loss, or current use of other kinds of apps for weight loss.	Weight
Hebden (2014)	Australia	RCT	41	15	22.6	3 months	BMI 24.00-31.99 kg/m^2^ with weight gain greater than 2 kg in the past 12 months, age 18-35 years, moderate intensity physical activity <60 min/day, SSB intake of at least 1 L weekly, fruit intake of less than two servings daily, vegetable intake of less than five servings daily, or at least two energy-dense takeaway meals weekly.	Inability to receive SMS messages or no regular internet access, a diet required for medical reasons, medical conditions that influence body weight or ability to comply with the intervention, intake of medications or herbal preparations that might influence body weight, enrollment in weight loss programs, pregnancy, or plan for pregnancy in the next 3 months.	Weight, BMI, and PA
Smith (2014)	Australia	RCT	361	100	12.7	7 months	Male students in their first year at the study schools completed a short screening questionnaire.	NR^f^	BMI and waist circumference
Glynn (2014)	Ireland	RCT	139	32	44	2 months	Age >16 years and active use of an Android smartphone.	No android smartphone, acute psychiatric illness, pregnancy, or inability to undertake moderate exercise.	Weight, BMI, and PA
Brindal (2013)	Australia	RCT	58	0	42	2 months	BMI >25 kg/m^2^ and ability to measure weight at home.	Medical conditions that are likely to interfere with the ability to undertake the meal replacement program (eg, pregnancy, breastfeeding, active cancer, gastrointestinal disorders, and type 1 diabetes).	Weight
Carter (2013)	UK	RCT	128	23.3	41.2	6 months	BMI ≥27 kg/m^2^, age 18-65 years, and willingness to commit the necessary time and effort to the study. Ability to read and write in English, ability to access the internet, and willingness to be randomized to one of three groups.	Pregnancy, breast feeding, plan for pregnancy, use of antiobesity medication or medication/insulin for diabetes, surgery for weight loss, and use of the antidepressant sertraline.	Weight and BMI
Allen (2013)	USA	RCT	35	22.1	44.9	6 months	Age 21-65 years, BMI 28-42 kg/m^2^, ownership of an iPhone or Android phone, willingness to download the app to be used on their device.	History of myocardial infarction, angina, coronary artery bypass graft surgery, percutaneous transluminal coronary angioplasty, congestive heart failure, and diabetes. Current participation in other weight loss programs, pregnancy, plan for pregnancy in the next 6 months, use of weight loss medications, and history of psychiatric illness, alcohol, or substance abuse within the past 12 months.	Weight, BMI, and waist circumference
McGrievy (2011)	USA	RCT	96	24.7	44	6 months	Age 18-60 years and BMI 25-45 kg/m^2^.	Smoking, unstable medical status, uncontrolled thyroid condition, inability to attend the three monitoring visits or improve the walking status, psychiatric illness, alcohol consumption, drug dependency, eating disorders, enrollment in another weight loss program, pregnancy, breast feeding, and plan for pregnancy within the next 6 months.	BMI and PA
Li (2010)	South Korea	CCS^g^	36	NR	28.5	6 weeks	Different ages and blood groups because of individual lifestyle and health effects according to blood group and the requirement of various amounts of calories based on gender.	NR	Weight and BMI

^a^RCT: randomized controlled trial.

^b^SSB: sugar-sweetened beverage.

^c^PA: physical activity.

^d^MPA: moderate physical activity.

^e^VPA: vigorous physical activity.

^f^NR: not reported.

^g^CCS: case-control study.

**Table 2:** Descriptions of baseline, interventions, apps, and findings of the included studies.

First author (year)	Baseline variables	Intervention type	App description	Control group treatment	Difference of the intervention group, mean (SD)	Difference of the control group, mean (SD)	Inference	Recommendation
Patel (2019)	Age, gender, marital status, race/ethnicity, education, employment status, annual household income, body mass index category, self-monitoring of diet frequency, and type of smartphone	App, email, MyFitnessPal, mobile, and internet	Weight loss goal, calorie goal, self-monitoring of body weight, dietary intake, rea-time feedback, skill training, and reminder of the goal	Self-regulation, email, and action plans via weekly email	−1.8 (1.53)	−2.55 (1.11)	The mobile app is an effective intervention for clinically meaningful weight loss.	Stand-alone digital health treatments may be a viable option for those looking for a lower intensity approach.
Farinelli (2016)	Age, gender, weight status, BMI, WHO-5 score, SES^a^, ethnic background, education, fruit, vegetable, SSB^b^, take-out meals, and physical activity	Mobile app, email, online weight tracker, physical activity planner, a blog facility for communication, and printable eating chart	Smart mobile apps for education and self-monitoring	Four text messages, one on each key behavior, and a two-page handout based on dietary guidelines.	−3.8 (4.9)	−0.80 (3.7)	The mHealth intervention has the potential to reduce weight and improve physical activity.	Replication of trials and widespread adoption of this model are needed.
Partridge (2015)	Age, gender, SES, ethnicity, education level, and weekly income	App, text messages, email, internet forum, a community blog, and usual care.	Educational program and self-monitoring	Mailed two-page handout, four text messages, and access to a website	−1.9 (2.84)	0.2 (2.99)	The app has huge potential for preventing weight gain with modest weight loss. It also helps to improve lifestyle behaviors.	Implementation of a large-scale study is needed.
Laing (2014)	Gender, self-reported race, education, annual income, and type of smartphone	App and usual care plan	MyFitnessPal app	Counseling and one-page educational handout for eating plan	−0.03 (4.64)	0.27 (4.64)	The app was an effective tool for reducing weight.	NR^c^
Hebden (2014)	Age, gender, SES, education, work history, lives with parents, and English proficiency	App, text messaging, email, internet forum, and usual care	Four types of behavior plans	10-page printed book	−1.6 (3)	−1.4 (3.18)	The app provided short-term positive changes in weight, nutrition, and physical activity.	More studies are needed to explore engagement and personalized support
Smith (2014)	Age, English language, cultural background, socioeconomic position, weight, height, BMI, weight status, and waist circumference	App, parent newsletters, seminars, spot sessions, lunchtime physical activity monitoring, and teaching material	Fitness challenges, activity monitoring, and motivational messages	Traditional approaches	0.6 (1.21)	0.61 (1.07)	The app-based intervention helped to improve fitness, movement skill, and key weight-related behavior.	More studies require to capture objective data on app usage throughout the intervention period and find out the association. It is also important to add some features like gamification.
Glynn (2014)	Gender, age, systolic and diastolic blood pressure, weight, BMI, HADS^d^, EQ-VAS^e^, EQ-5D^f^, and daily step count	App and usual care plan	Accupedo-Pro Pedometer app	Education program about the benefits of physical activity and exercise	−2.2 (3.4)	−1.5 (4.3)	The mobile app–based intervention had a positive impact on weight loss	NR
Brindal (2013)	Weight and dietary status	App and celebrity slim program	Support apps like my meals, my weight, and my task	Only celebrity slim program	−2.9 (6.4)	−2.1 (1)	The app intervention was useful for weight loss and psychological changes.	Integrating more dynamic stage-based tailoring, as behavioral changes of individuals may further enhance similar apps in the future.
Carter (2013)	Age, weight, BMI, body fat, gender, race, smoking status, occupation, and education	App	Self-monitoring	Food diary and a calorie-counting book	−4.6 (5.2)	−2.9 (5.85)	The mobile app was an acceptable and feasible weight loss intervention	More studies are needed to investigate the cost of implementing a smartphone app intervention compared with other types of interventions
Allen (2013)	Age, weight, BMI, waist circumference, education, and marital status	App and intensive counseling	Lose it!	Comprehensive counseling	−5.4 (4)	−2.5 (4.1)	The app intervention had a positive impact on weight loss and contributed to behavioral changes.	Need to conduct a large-scale population-based study.
McGrievy (2011)	NR	App + podcast + twitter	Diet plan and physical activity monitoring	Podcast only	−2.57 (2.6)	−2.45 (4.39)	NR	NR
Li (2010)	Age, occupation, education, monthly income, smoking, drinking, and exercise history	Mobile app and usual care	Mobile apps that provided a personal diet profile based on gender and promoted knowledge about nutrition and physical activity	NR	−1.9 (2.3)	−0.9 (4.64)	Improved user satisfaction.	A more effective study to motivate participants and extend study duration is required.

^a^SES: socioeconomic status.

^b^SSB: sugar-sweetened beverage.

^c^NR: not reported.

^d^HADS: Hospital Anxiety and Depression Scale.

^e^EQ-VAS: EuroQol visual analogue scale.

^f^EQ-5D: EuroQol five-dimension scale.

### Assessment of the Risk of Bias

**Figure 2:**
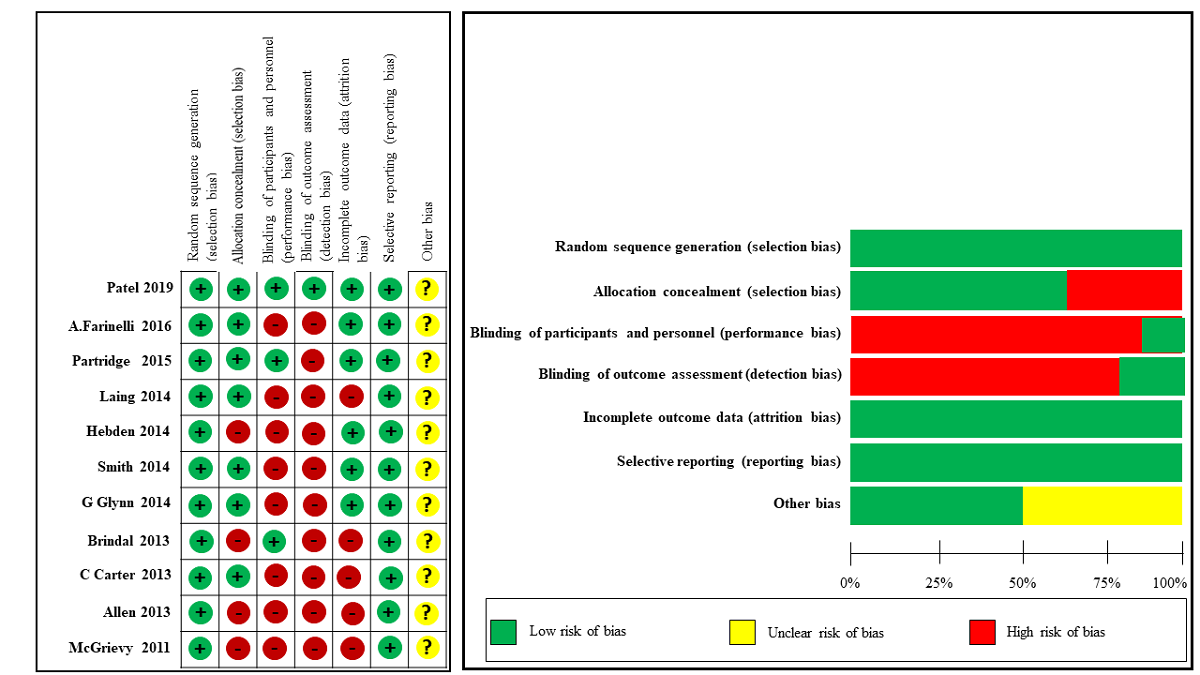
Risk bias assessment of the included studies.

Owing to the nature of mobile app interventions, participant blinding is not always feasible in trials. All the 12 studies reported random sequence generation that showed a low risk of bias. Overall, 10 out of the 12 studies were considered to constitute high-quality evidence. Only three studies had blinding of the outcome assessment, and two studies had blinding of the participants and personnel. A summary of the evaluation of the included studies is shown in Figure 2.

### Mobile App Intervention and Weight Loss

**Figure 3:**
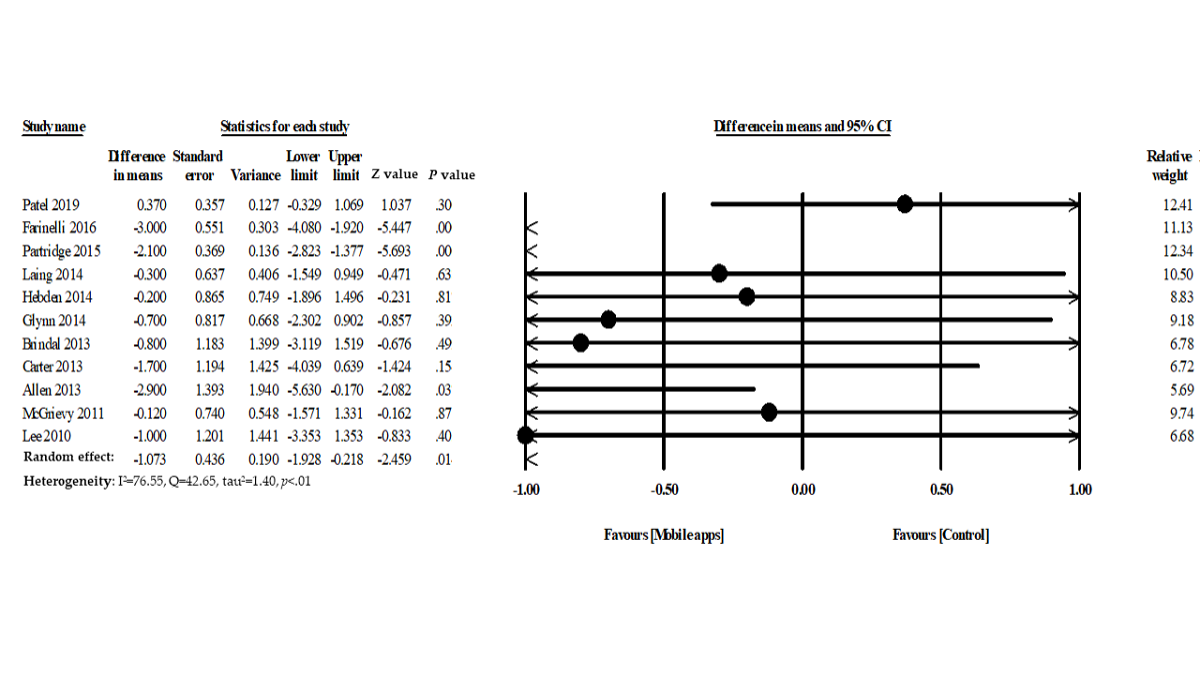
Forest plot of mobile phone app interventions and weight loss.

Eleven studies assessed the effectiveness of mobile phone app interventions for reducing body weight. Participants in the intervention group showed a decrease in their body weight (−1.07 kg, 95% CI −1.92 to −0.21) when compared with the control group (Figure 3). However, moderate heterogeneity was observed among the studies (heterogeneity I^2^=71.55%, Q=42.65, *P*=.01, τ^2^=1.40).

### Mobile App Intervention and BMI

**Figure 4:**
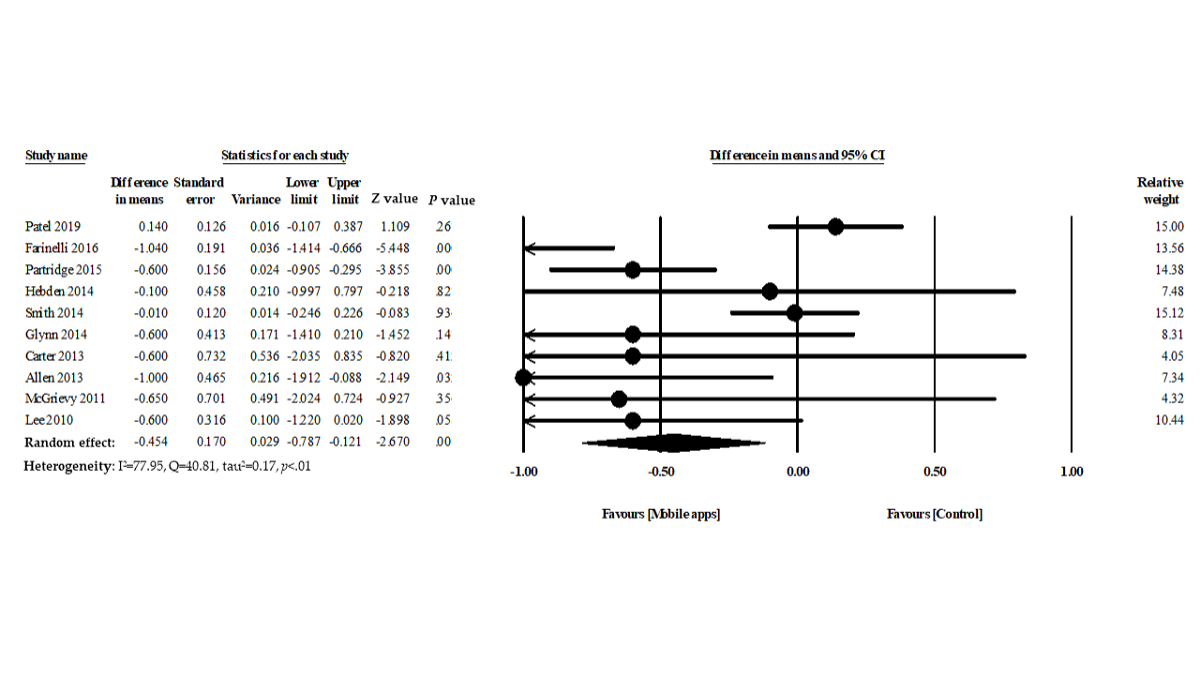
Forest plot of mobile phone app interventions and change in BMI.

A total of 10 studies evaluated the efficacy of mobile apps for BMI reduction. The overall pooled findings showed a significant difference in BMI between participants in the mobile phone app intervention group and control group (−0.45 kg/m^2^, 95% CI −0.78 to −0.12, *P*=.008, heterogeneity I^2^=77.95%, Q=40.81, τ^2^=0.17) (Figure 4).

### Mobile App Intervention and Physical Activity

**Figure 5:**
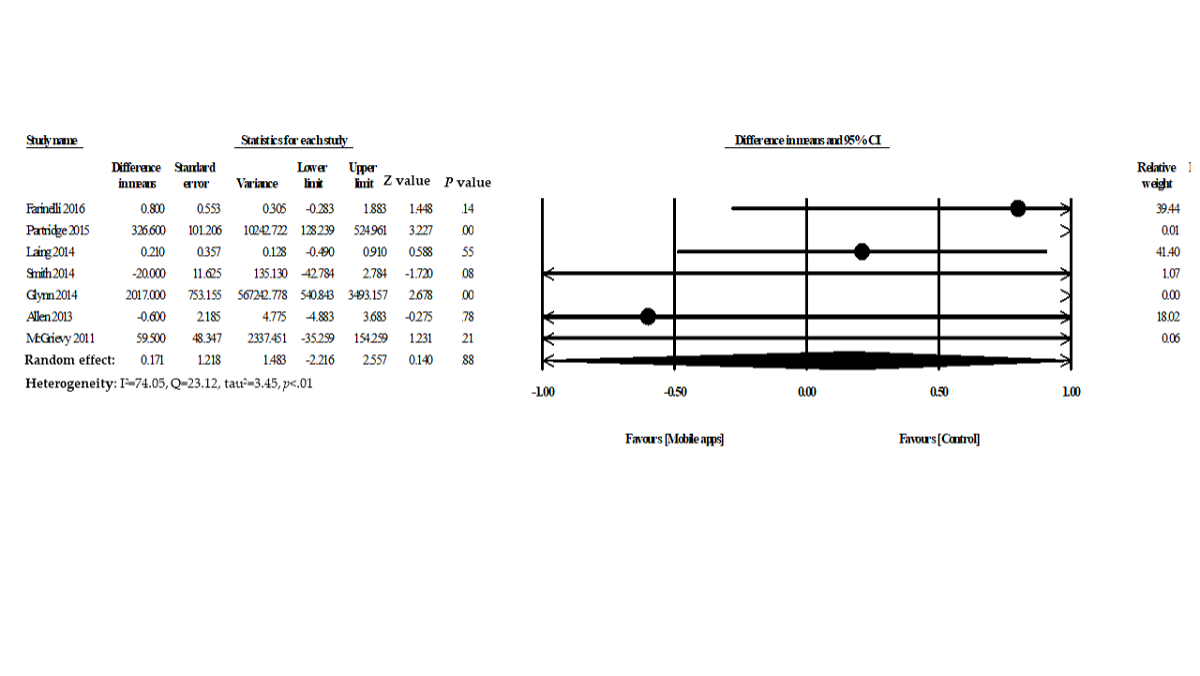
Forest plot of mobile phone apps for increased physical activity.

Seven studies assessed the effectiveness of mobile phone apps for increasing physical activity. The usability and effectiveness of mobile apps had promising but insignificant results (mean difference 0.17, 95% CI −2.21 to 2.55, *P*=.88). However, moderate heterogeneity was observed among the included studies (heterogeneity I^2^=74.05%, Q=23.12, and τ^2^=3.45) (Figure 5).

### Sensitivity Analysis

Five studies evaluated the effectiveness of mobile apps used ≤3 months for weight loss. Pooled findings indicated no significant difference in weight between the mobile app intervention group and the control group (−0.004 kg, 95% CI −0.79 to 0.80, *P*=.99). Six studies evaluated the effectiveness of mobile apps used >3 months for weight loss. Pooled findings showed a significant difference in weight between the mobile app intervention group and the control group (−1.63 kg, 95% CI −2.64 to −0.61, *P*<.002). In addition, the pooled findings from only the randomized controlled trials showed a significant difference in weight between the mobile app intervention group and the control group (−1.03 kg, 95% CI −2.05 to −0.025, *P*=.04) (Multimedia Appendix 3, Multimedia Appendix 4, and Multimedia Appendix 5).

### Publication Bias

**Figure 6:**
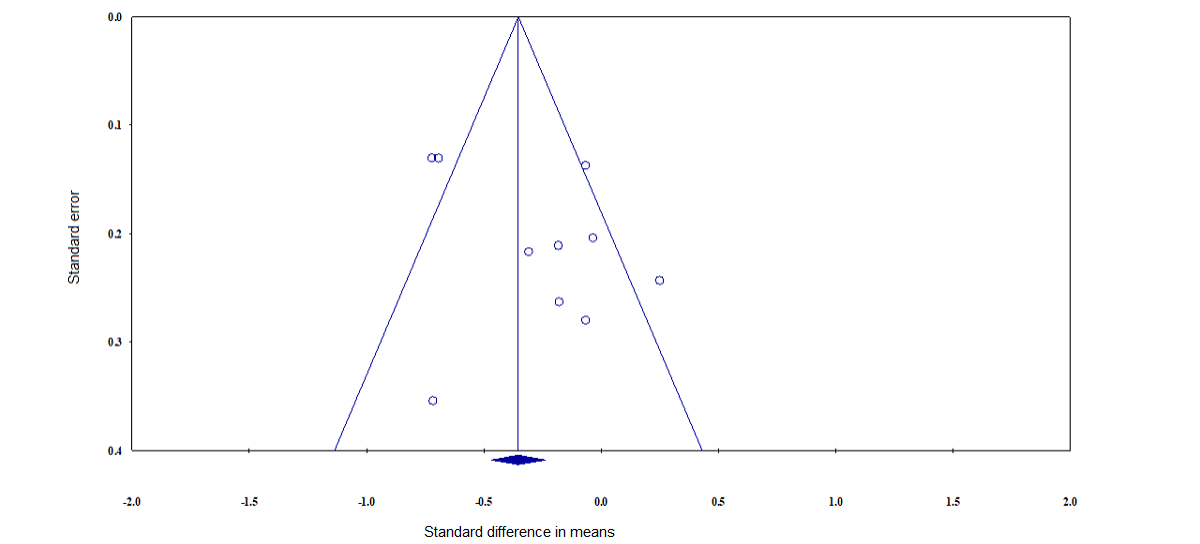
Funnel plot.

The meta-analysis of the observational studies had some sort of publication bias. Egger regression test was used to calculate the publication bias, and a funnel plot was drawn to visualize it. The funnel plot in Figure 6 shows no relevant publication bias.

## Discussion

### Principal Findings

This meta-analysis evaluated the effectiveness of mobile app interventions for weight management. The meta-analysis showed a small but significant reduction in body weight (−1.07 kg) and a reduction in BMI (−0.45 kg/m^2^). Our findings are more comprehensive across sensitivity analyses. Moreover, our findings showed that mobile app interventions promote additional positive health benefits through the maintenance of BMI and increased physical activity from baseline. The ubiquitous use of a mobile app intervention in any age group may therefore have great clinical value when compared with traditional interventions. A previous systematic review and meta-analysis reported pooled effects of app interventions compared with controls of −1.04 kg for weight loss and −0.45 kg/m^2^ for BMI [14]. The magnitude of the mobile app intervention effect in our updated meta-analysis suggests that the use of mobile app interventions is effective for promoting body weight management following an initial weight loss when compared with other interventions.

### Public Health Implications

Rising obesity and physical inactivity are associated with chronic diseases and increased health costs [31]. To reduce the health care burden, researchers have already pointed out the importance of effective health communication. Digital technology provides fast and interactive communication that is easy to use and cost-effective. Mobile phones have emerged as a potential medium for interventions to assist people in maintaining health, and they have shown promising results for weight loss and increased physical activity [32]. However, a mobile app can be tailored to the individual, and information could be delivered in a more effective way that may be more realistic and feasible than conventional ways to deliver information. Several studies have investigated the efficacy of weight management using text messaging interventions. A previous meta-analysis that included 14 studies with an intervention period ranging from 1 month to 2 years found that text messaging interventions can promote weight loss (−2.56 kg, 95% CI −3.46 to −1.65) [33].

Several studies have previously highlighted targeting behavioral change techniques that include dietary self-monitoring and reporting, behavior reinforcement through motivational messages, social support, setting and evaluating various goals, and setting reminders, and they are all key components to reduce and maintain weight successfully [34]. The use of mobile apps helps to improve self-awareness, provides valuable information, and can be an early indicator of health-related issues; this information and support can help to spur positive behavioral changes [35]. Results from this study suggest that even in the short term (<6 months), mobile app interventions provide a generally positive effect for reducing weight and maintaining BMI. In this meta-analysis, mobile apps were related to weight loss goals, calorie goals, self-monitoring of body weight, dietary intake, real-time feedback, educational content, behavioral change plans, nutrition, physical activity, several types of trainings, and reminders of goals. Moreover, the intervention types included apps, email, internet systems, online weight trackers, physical activity planners, blog facilities for communication, internet forums, parental newsletters, seminars, spot sessions, podcasts, twitter, and printable eating charts, which were all deemed to be effective for reducing weight and maintaining BMI.

### Limitations

This meta-analysis shows that a mobile app intervention has a variety of uses in reducing weight and maintaining BMI with immense benefits. However, there are several limitations in this study that we need to address. First, the findings of this study should be interpreted with caution considering the sample sizes of the included studies and considering the studies that reported on short-term periods. To increase the generalizability of our findings, a larger sample size with a longer follow-up period (at least 1 year) in diverse racial or ethnic settings is needed. Second, although heterogeneity among the studies was not high, the results were nevertheless based on a relatively small number of studies. A small number of studies also prevented us from conducting a meta-regression analysis to evaluate other factors for reducing weight and maintaining BMI.

### Conclusions

The findings of this study suggest that mobile app interventions appear to be feasible and acceptable for reducing weight, maintaining BMI, and increasing physical activity, although the overall effects might be relatively modest. However, the course averages hide some variation; therefore, such interventions are highly successful in some people and completely ineffective in some people. Public awareness of safety and the benefits of weight management and physical activity should be promoted, and more studies with a larger sample and longer follow-up are needed to evaluate the potential role of a mobile phone app intervention.

## Appendix

Multimedia Appendix 1 PRISMA checklist.

Multimedia Appendix 2 Search words.

Multimedia Appendix 3 Intervention duration ≤3 months.

Multimedia Appendix 4 Intervention duration >3 months.

Multimedia Appendix 5 Only randomized controlled trials.
