# Supplementary figures and images for: Use of Mobile Phone App Interventions to Promote Weight Loss: Meta-Analysis
Source: JMIR Mhealth Uhealth. 2020 Jul 22;8(7):e17039. doi: 10.2196/17039 (PMC7407260; doi:10.2196/17039)

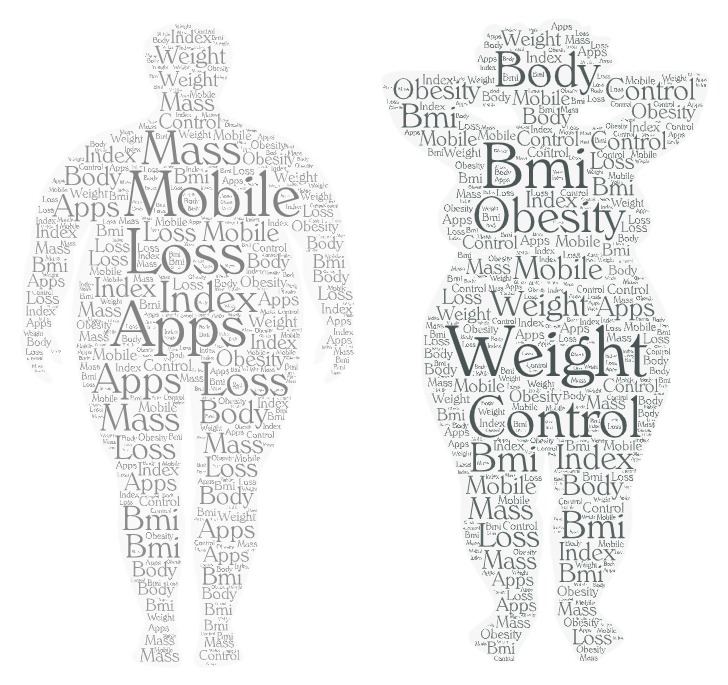


**Supplementary Figure S1:** Search words

Supplement: Multimedia Appendix 2 [file mhealth_v8i7e17039_app2.docx]

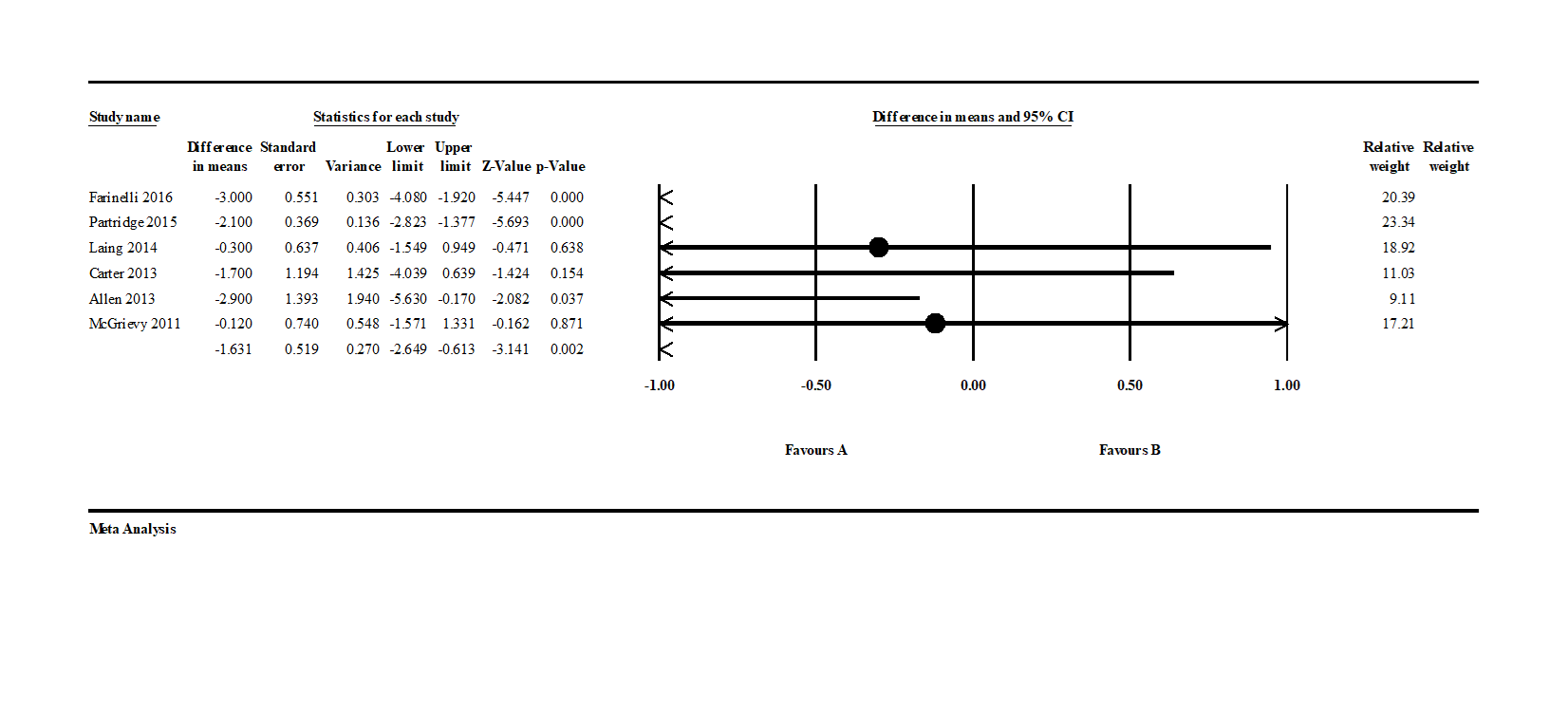


**Supplementary Figure S3**: Intervention duration more > 3 months

Supplement: Multimedia Appendix 4 [file mhealth_v8i7e17039_app4.docx]
