# Supplementary material for: Use of Mobile Phone App Interventions to Promote Weight Loss: Meta-Analysis
Source: JMIR Mhealth Uhealth. 2020 Jul 22;8(7):e17039. doi: 10.2196/17039 (PMC7407260; doi:10.2196/17039)
Supplement: Multimedia Appendix 5 [file mhealth_v8i7e17039_app5.docx]

**Supplementary Figure S4**: Only randomized controlled trials
